# Early Detection of Nasopharyngeal Carcinoma

**DOI:** 10.1155/2011/638058

**Published:** 2011-06-08

**Authors:** Keiji Tabuchi, Masahiro Nakayama, Bungo Nishimura, Kentaro Hayashi, Akira Hara

**Affiliations:** Department of Otolaryngology, Graduate School of Comprehensive Human Sciences, University of Tsukuba, 1-1-1 Tennodai, Tsukuba 305-8575, Japan

## Abstract

Nasopharyngeal carcinoma (NPC) is a unique disease with a clinical presentation, epidemiology, and histopathology differing from other squamous cell carcinomas of the head and neck. NPC is an Epstein-Barr virus-associated malignancy with a marked racial and geographic distribution. Specifically, it is highly prevalent in southern China, Southeast Asia, and the Middle East. To date, most NPC patients have been diagnosed in the advanced stage, but the treatment results for advanced NPC are not satisfactory. This paper provides a brief overview regarding NPC, with the focus on the early detection of initial and recurrent NPC lesions.

## 1. Introduction

Nasopharyngeal carcinoma (NPC) is a nonlymphomatous squamous cell carcinoma that occurs in the epithelial lining of the nasopharynx. This neoplasm shows varying degrees of differentiation and is frequently seen in the pharyngeal recess (Rosenmüller's fossa), posteromedial to the medial crura of the Eustachian tube opening in the nasopharynx [1].

NPC is a distinct form of head and neck cancer that differs from other malignancies of the upper aerodigestive tract in terms of its etiology, epidemiology, pathology, clinical presentation, and response to treatment [2]. Outside of endemic areas in Southeast Asia, NPC is rare, occurring in less than 1/1,000,000 people [3]. In North America, NPC accounts for approximately 0.2% of all malignancies, with approximately 0.5–2 cases per 100,000 males and about one-third of that in females [4–6]. The incidence of NPC reportedly remains high among Chinese people who have emigrated to Southeast Asia or North America, but is lower among Chinese people born in North America than in those born in Southern China [7, 8]. This finding suggests that genetic as well as environmental factors play a role in the cause of the disease [9]. 

The mainstay of NPC treatment is radiotherapy, but treatment results for advanced NPC is not satisfactory. The focus of this review is to provide an overview of NPC, especially the recent insights regarding early detection of NPC.

## 2. Epidemiology and Etiology

 NPC is a relatively rare malignancy in most parts of the world. It accounts for 2% of all head and neck squamous cell carcinomas, with an incidence of 0.5 to 2 per 100,000 in the United States [10]. However, it is endemic in many geographical regions, including Southern China, Southeast Asia, Japan, and the Middle East/North Africa [10, 11]. Ho [12] reported that NPC is the third most common malignancy among men, with an incidence of between 50 per 100,000 in the Guangdong Province of Southern China. Emigration from high- to low-incidence areas such as the United States and Canada reduces the incidence of NPC in first-generation Chinese, but it still remains at seven-times the rate in Caucasians [8]. 

NPC presents as a complex disease caused by an interaction between chronic infection with oncogenic gamma herpesvirus Epstein-Barr virus (EBV) and environmental and genetic factors, involving a multistep carcinogenic process [10]. EBV exists worldwide, infecting over 95% of the global adult population [13]. In Hong Kong, 80% of children are infected by 6 years of age, and almost 100% have seroconverted by 10 years of age [14]. Although primary EBV infection is typically subclinical, the virus is associated with the later development of several malignancies, including NPC [11]. It is transmitted by saliva, and its primary infection occurs during childhood with replication of the virus in the oropharyngeal lining cells, followed by a latent infection of B lymphocytes (primary target of EBV). Elevated titers of EBV-associated antigens (especially of IgA class), a latent EBV infection indentified in neoplastic cells of virtually all cases of NPC, and the clonal EBV genome consistently detected in invasive carcinomas and high-grade dysplastic lesions suggest a critical role of EBV in the pathogenesis of NPC in endemic areas [10]. 

Nonviral exposure associated with the risk of NPC involves the consumption of salt-preserved fish, a traditional staple food in several NPC-endemic areas [11]. In studies of Chinese populations, the relative risk of NPC associated with weekly consumption, compared with no or rare consumption, generally ranged from 1.4 to 3.2 per 100,000 whereas that for daily consumption ranged from 1.8 to 7.5 [15–22]. Salt-preserved foods are a dietary staple in all NPC-endemic populations [23]. Thus, this dietary staple pattern may explain part of the international distribution of NPC incidence. The carcinogenic potential of salt-preserved fish is supported by experiments in rats, which develop malignant nasal and nasopharyngeal tumors after salted fish consumption [18, 24, 25]. The process of salt preservation is inefficient, allowing fish and other foods to become partially putrefied. As a result, these foods accumulate significant levels of nitrosamines, which are known carcinogens in animals [23, 26, 27]. Salt-preserved fish also contain bacterial mutagens, direct genotoxins, and EBV-reacting substances [28–30], any or all of which could also contribute to the observed association. However, there have been no prospective studies of NPC risk associations with salt-preserved fish consumption, or virtually any other environmental exposure, in endemic areas.

 Several associations have been described between the frequency of human leukocyte antigen (HLA) class I genes in certain populations and the risk of developing NPC. For example, increased risk of NPC was observed in individuals with the *HLA-A2* allele, particularly *HLA-A*0207 [31]. Recent genome-wide association studies confirmed involvement of HLA molecules in NPC generation [32, 33]. Cellular gene alterations also contribute to development of NPC, especially inactivation of tumor suppressor genes, SPLUNC1, UBAP1, BRD7, Nor1, NGX6, and LTF [34].

## 3. Pathology

In 1978, the histological classification guideline proposed by the World Health Organization (WHO) categorized NPC into three groups: type 1 (keratinizing squamous cell carcinoma), type 2 (nonkeratinizing carcinoma), and type 3 (undifferentiated carcinoma). The 1991 WHO classification of nasopharyngeal carcinoma divided them into two groups: squamous cell carcinoma (keratinizing squamous cell carcinoma, type 1 of the former classification), and nonkeratinizing carcinoma (types 2 and 3 of the former classification combined into a single category). Nonkeratinizing carcinoma was further subdivided into differentiated and undifferentiated carcinomas [35]. This classification is more applicable for epidemiological research and has also been shown to have a prognostic significance. Undifferentiated carcinomas have a higher local tumor control rate with treatment and a higher incidence of distant metastasis than do differentiated carcinomas [36, 37]. 

 Published data indicate a higher proportion of keratinizing squamous cell carcinoma among all NPC in nonendemic compared with endemic areas. Some studies reported that squamous cell carcinoma accounts for approximately 25% of all NPC in North America, but only 1% in endemic areas; whereas undifferentiated carcinoma accounts for 95% of all cases in high-incidence areas, but 60% of cases in North America [9, 10, 38].

## 4. Initial Treatment

 Radiotherapy is the mainstay of treatment for NPC. Typical radiation fields encompass the adjacent skull base and nasopharynx. Fields are bilaterally directed and include the retropharyngeal lymphatic drainage pathway. The control rate on conventional radiotherapy is 75 to 90% in T1 and T2 tumors, and 50 to 75% in T3 and T4 tumors. Because of the high incidence of occult cervical node metastasis, prophylactic neck radiation is recommended even in N0 cases [39]. The control of cervical nodal regions is achieved in 90% of N0 and N1 cases, and about 70% of N2 and N3 cases [40]. It is mandatory to keep the treatment schedule because interrupted or prolonged treatment reduces the benefits of radiotherapy [41].

 Recent studies have suggested that addition of chemotherapy to radiotherapy improves the treatment results in patients with nasopharyngeal carcinoma. Phase III randomized intergroup study 0099 showed that patients treated with radiation alone had a significantly lower 3-year survival rate than those receiving radiation with cisplatin and 5-fluorouracil chemotherapy [42]. A meta-analysis of chemotherapy for NPC conducted by Baujat et al. [43] employed an individual patient data design. They reported a definite improvement of the 5-year survival rate due to the addition of chemotherapy (56% with radiotherapy alone versus 62% with chemoradiotherapy). In addition to these findings, other phase III or meta-analysis studies also reported the superiority of concurrent chemoradiotherapy versus radiotherapy alone [44–46]. The above-described reports suggest the benefits of the addition of chemotherapy, especially in advanced NPC cases. However, there is still debate on the effectiveness of the addition of chemotherapy, and issues regarding the addition of adjuvant chemotherapy are even more controversial [40].

## 5. Early Detection of Nasopharyngeal Carcinoma

 Wei and Sham [9] divided symptoms presented by NPC patients into four categories: (1) symptoms caused by the presence of a tumor mass in the nasopharynx (epistaxis, nasal obstruction, and discharge), (2) symptoms associated with dysfunction of the Eustachian tube (hearing loss), (3) symptoms associated with the superior extension of the tumor (headache, diplopia, facial pain, and numbness), and (4) neck masses. Because symptoms related to NPC in the early stage are usually nonspecific, most NPC patients are diagnosed in the advanced stage. As treatment results for NPC are not satisfactory in the advanced stage, early diagnosis and appropriate management are important to achieve favorable treatment results. The development of a good primary NPC screening protocol may thus contribute to the early detection and improve the treatment outcome.

 The endemic form of NPC is associated with EBV, although the exact role of EBV in the pathogenesis of NPC remains unclear. IgA antibody titers to EBV viral capsid antigen (EBV-IgA-VCA) and EBV early antigen (EBV-EA) in immunofluorescent assays may be used for the serologic screening of NPC [47, 48]. In recent years, enzyme-linked immunosorbent assays (ELISA) employing purified recombinant EBV antigens are increasingly advocated in place of traditional immunofluorescent assays [49]. These tests frequently precede the appearance of NPC and serve as tumor markers of remission and relapse [50, 51]. Ji et al. [52] monitored EBV IgA antibody levels of NPC cases in a prospective manner. They confirmed that elevation of the EBV antibody levels preceded the clinical onset of NPC. They also reported that there is a window of about 3 years preceding the clinical onset, when the antibody level is elevated and maintained at high levels [53]. However, none of these serologic screening tests appear satisfactory to date because of low-level sensitivity or specificity. Detection of the EBV gene in nasopharyngeal swabs from symptomatic patients has been shown to be highly predictive of symptomatic NPC [54, 55]. 

Proteomic approaches have been applied for the analysis of malignant neoplasms. For practical usage in tumor screening, biomarkers should be measurable in body fluid samples [55]. Recently, Wei et al. [56] analyzed serum samples from patients with NPC employing proteomic analysis. In their report, four protein peaks at 4,097, 4,180, 5,912, and 8,295 daltons (Da) discriminated NPC patients with a sensitivity of 94.5% and specificity of 92.9%. Furthermore, Chang et al. [55] reported that the use of a three-marker panel (cystatin A, MnSOD, and MMP2) could contribute to improved NPC detection. Other potential markers for the diagnosis of NPC include Galectin-1, fibronectin, Mac-2 binding protein, and plasminogen activator inhibitor 1 [57, 58]. There is a possibility that the incorporation of these tests in the routine screening of NPC may enhance its early detection. 

The importance of clinical syndromes, history, and clinical examination for helping the early diagnosis of NPC could not be ignored. Individuals with acquired immunodeficiency syndrome (AIDS) manifest an increased risk of NPC [59]. The most common presenting complaint is a painless upper neck mass or masses. Any adult presenting with unexplained unilateral serous otitis media should be carefully examined to rule out NPC. Endoscopy plays a key role in detecting the early NPC lesions, and endoscopic biopsy enables their definitive diagnosis. Early lesions usually occur on the lateral wall or roof of the nasopharynx. Vlantis et al. [60] reported an objective endoscopic score of abnormality of nasopharynx to predict the likelihood of NPC. However, clinicians should keep in mind the fact that detection of NPC is sometimes difficult with endoscopy. Endoscopic findings may be subtle in early NPC lesions: only slight fullness in the Rosenmüller's fossa, or a small bulge or asymmetry in the roof. When NPC is strongly suspected, considering early diagnosis of NPC, appropriate imaging examinations and/or biopsy of the nasopharyngeal mucosa are recommended even if the mucosal surface exhibits normal appearance. 

 Careful attention should be paid when MRI is conducted for a patient with unilateral serous otitis media (stasis of secretions in unilateral middle ear) or cervical lymph node adenopathy. Most NPC cases originate in Rosenmüller's fossa. Obstruction of the pharyngeal orifice of the Eustachian tube results in serous otitis media. Approximately 70% of NPC patients initially present with neck masses, and 60 to 96% of NPC patients exhibited cervical lymph node adenopathy at the time of presentation [61–63]. Neck masses are usually observed in the upper neck [40]. T1 tumors, confined to the nasopharynx, may be clinically occult, and also may be difficult to differentiate from the normal mucosa on a CT scan and MRI. However, such small tumors are usually readily evident by their less intense enhancement by gadolinium than the normal nasopharyngeal mucosa [64]. Furthermore, MRI may help to depict subclinical cancers missed at endoscopy [65]. It has been suggested that MRI is superior to 18-fluoro-2-deoxyglucose (FDG) positron emission tomography (PET) for the assessment of locoregional invasion and retropharyngeal nodal metastasis. PET is not suitable for detecting small retropharngeal nodes or for distinguishing retropharyngeal nodes from adjacent primary tumors [66].

## 6. Early Diagnosis of Recurrent Nasopharyngeal Carcinoma

 To date, the modalities commonly used in the followup of patients with NPC include clinical examinations and imaging studies. Inspection with a flexible fiberscope plays a primary role in followup examinations. However, mucosal reactions to radiotherapy make it difficult to find early recurrent lesions. Secretions and crust covering the nasopharyngeal mucosa also hamper the early detection of local recurrence. In addition, the detection of submucosal or deep-seated recurrent lesions is difficult with fiberscopic examinations. If recurrent NPC lesions can be diagnosed properly and in a timely manner, these lesions may be treated by chemotherapy, reirradiation, such as further conventional external beam radiotherapy, brachytherapy, and stereotactic radiotherapy, or surgery [9]. Regarding surgery, conventional nasopharyngectomy for recurrent NPC lesions can still result in serious complications. However, early recurrent lesions (such as rT1 lesions) may be effectively treated with laser nasopharyngectomy [67]. Diagnostic uncertainty may result in delayed treatment, which reduces the life expectancy of patients with recurrent NPC lesions.

 Narrow-band imaging (NBI) is a novel technique that enhances the diagnostic sensitivity of endoscopes for characterizing tissues using narrow-bandwidth filters in a sequential red-green-blue illumination system. Superficial mucosal carcinoma lesions, which are rarely detected using conventional endoscopy, can be observed with NBI by viewing the nonangiogenetic, microvascular proliferation pattern [68, 69]. Recently, Lin and Wang [69] applied this technique to the detection of early recurrent mucosal lesions of NPC. They reported that early recurrent lesions of NPC after radiotherapy were successfully detected by NBI coupled with conventional endoscopy.

 Regarding imaging studies after initial treatment, CT and MRI are widely used for the detection of recurrent lesions. Generally, MRI is superior to CT in the detection of soft tissue abnormalities. The baseline MRI study is often conducted 2 to 3 months after termination of the initial treatment. After the baseline evaluation, close evaluation is recommended with further imaging followup every 3 to 6 months for the first 2 years posttreatment [63]. Edema induced by radiotherapy may be noted in the initial imaging studies. However, any signal abnormalities in the nasopharynx on MRI should be stable or reduced in this followup period. After 2-year followup without evidence of recurrence, the imaging interval is extended to be every 6 to 12 months [63]. Recently, the effectiveness of FDG-PET in the detection of residual or recurrent NPC lesions has been reported from several institutes. FDG-PET is increasingly being used for detection of recurrent lesions in many types of tumor. PET is reportedly useful to distinguish recurrent NPC tumors from postirradiation changes, such as tissue necrosis, fibrosis, and edema [70–73]. Liu et al. [74] reported that sensitivities of CT, MRI, and PET for the detection of residual or recurrent NPC lesions were 76, 78, and 95%, respectively. These findings suggest that PET can be a useful tool for the detection of recurrent NPC lesions. However, there are also some limitations regarding the use of PET for the detection of early recurrent NPC lesions. FDG uptake was increased by inflammatory reactions in the early period after radiotherapy [74]. Furthermore, a recent cost-based analysis suggested that it is most cost-effective to perform PET if MRI results are unclear [75].

## 7. Conclusions

 NPC detection in the early stage is often difficult because the symptoms are not specific. EBV-related serologic tests are used as screening tools in high-risk populations, although the screening tests available in daily clinics are not satisfactory. Molecular biomarkers are under examination as a new tool for the detection of early NPC lesions. Regarding imaging modalities, MRI seems suitable for the detection of early lesions, and the routine use of PET for the initial diagnosis of NPC does not seem to be justified. The early diagnosis of recurrent or residual NPC lesions is also challenging. Postradiation mucosal reactions make a precise diagnosis difficult. PET is useful in distinguishing recurrent NPC regions if MRI findings are not definitive. NBI may also be useful in detecting early recurrent mucosal lesions. In addition to the new diagnostic modalities, improvement in the awareness of physicians and the general population regarding this carcinoma undoubtedly contributes to the earlier detection of the disease.
